# Pathophysiological mechanisms of Postural Orthostatic Tachycardia Syndrome analyzed by means of hemodynamics

**DOI:** 10.1371/journal.pone.0327236

**Published:** 2025-07-02

**Authors:** Liuchuang Wei, Heming Cheng, Suihai Chen, Jifeng Dai, Gen Li, Dongfang Ding, Xue Zhang, Ke Zhang, Jianyun Li, Jie Hou

**Affiliations:** 1 Department of Mechanics, Kunming University of Science and Technology, Kunming, PR China; 2 Department of Hydraulic Engineering, Kunming University of Science and Technology, Kunming, PR China; Tokai University School of Medicine, JAPAN

## Abstract

Postural Orthostatic Tachycardia Syndrome is characterized by an excessive increase in heart rate during postural changes without significant blood pressure alterations, often accompanied by symptoms such as dizziness, fatigue, and palpitations. While prior studies have explored potential mechanisms, the precise etiology and pathological basis of Postural Orthostatic Tachycardia Syndrome remain unclear, leading to treatments focused on symptom management rather than addressing root causes. This study employs a hemodynamic fluid-structure interaction model (using existing clinical data) to investigate the roles of hypovolemia, vascular dysfunction, and autonomic nervous system dysregulation in Postural Orthostatic Tachycardia Syndrome pathogenesis. Computational results revealed that hypovolemia reduces cerebral blood flow by approximately 100 mL/min due to a 30% decrease in blood volume, while vascular dysfunction—marked by a 50–100% increase in arterial stiffness—further diminishes cardiac output and cerebral perfusion. These conditions trigger compensatory tachycardia through autonomic feedback mechanisms. Our findings demonstrate that an insufficient cerebral blood supply, driven by hypovolemia and impaired vascular compliance, combined with autonomic dysregulation, underlies the hallmark tachycardia in Postural Orthostatic Tachycardia Syndrome. This mechanistic insight provides a foundation for targeted therapeutic strategies, emphasizing the need to address blood volume management, vascular elasticity, and autonomic balance to improve clinical outcomes.

## 1 Introduction

Postural Orthostatic Tachycardia Syndrome (POTS) is a medical condition distinguished by an excessive elevation of heart rate (HR) and negligible alteration in blood pressure (BP) during positional transitions, especially when moving from a lying position to a standing one. POTS predominantly affects young women, and epidemiological data indicates a prevalence of approximately 0.2% in the general population [1,2]. The clinical significance of POTS lies in its profound impact on patients’ quality of life. The condition is characterized by a diverse array of symptoms associated with autonomic dysfunction, including palpitations, dizziness, fatigue, and cognitive impairment, which may lead to limitations in daily activities, severely affecting their ability to work and socialize [2–4]. Recent studies have centered on possible connections among hormone fluctuations, genetic factors, lifestyle habits, other diseases, and POTS [3,5]. Unlike neurally mediated syncope (vasovagal syncope), which involves transient hypotension and fainting, POTS is characterized primarily by a sustained tachycardia without significant BP drop [6].

Hypovolemia is one of the main pathological features in POTS [3,4,6,7]. POTS patients frequently experience hypovolemia, which may be caused by insufficient fluid intake and excessive fluid loss. Studies have shown that when POTS patients are in the orthostatic position, their venous return is reduced. This leads to a decrease in cardiac preload, and as a result, the HR increases significantly to maintain cerebral blood supply [2,8]. In addition, hypovolemia is closely associated with sympathetic nervous system (SNS) activation, which exacerbates the symptoms of tachycardia [9].

Vascular dysfunction plays a significant role in the clinical presentation of POTS [10,11]. The arterial vasoconstrictive capacities of POTS patients are often compromised, leading to difficulties in BP regulation during postural changes. Research indicates that insufficient venous return in patients with POTS further exacerbates tachycardia [2,12]. The association between vascular dysfunction and heightened SNS excitability has been postulated, with potential links to vascular endothelial dysfunction and *β*-receptor hypersensitivity [12]. A comprehensive understanding of vascular function in POTS patients is therefore paramount for the development of effective treatment plans.

The ANS plays a critical role in the pathogenesis of POTS [3,6,13]. Patients with POTS often exhibit abnormalities in ANS function, including dysregulation of SNS and parasympathetic nervous system. Investigations have demonstrated that patients with POTS commonly present orthostatic tachycardia owing to SNS hyperactivation [2–4]. These patients typically show elevated SNS activity and diminished parasympathetic activity in the evaluation of autonomic function testing, resulting in a substantial increase in HR [7,13]. Additionally, epinephrine and norepinephrine secreted by the adrenal medulla can activate the *β*_1_ and *β*_2_ adrenergic receptors in the heart, producing a typical sympathetic response and increasing HR [14].

Research into the pathomechanisms of POTS faces several challenges. Although standardized diagnostic criteria for POTS exist (e.g., a ≥ 30 bpm HR increase on standing), patient heterogeneity complicates efforts to standardize research and develop targeted treatments [15]. Clinically, POTS and neurally mediated syncope are associated with autonomic nervous system dysfunction; however, POTS is characterized primarily by a marked increase in heart rate upon standing, whereas neurally mediated syncope typically manifests as syncope accompanied by an abrupt decline in heart rate. Additionally, POTS and orthostatic hypotension are often misdiagnosed due to overlapping triggers (such as postural changes, dehydration, dietary influences), overlapping symptoms (e.g., dizziness, fatigue, palpitations), and the inherent complexity of diagnostic criteria [13,16]. Second, current treatment approaches are largely empirical and focus on symptom relief rather than mechanism-oriented strategies [17,18]. Furthermore, POTS manifestations may vary among individuals, complicating the development of a universally applicable model to explain all clinical features [19,20].

Recently, Cheng et al. presented a fluid-structure interaction model of blood circulation to theoretically analyze blood flow in the circulation system [21,22]. This model encapsulates the coupling effects between blood circulation and the intrinsic properties of blood vessels, offering a novel perspective on POTS pathogenesis. Compared to previous models (e.g., Windkessel-type [23] or lumped-parameter [24] models), this new model uniquely integrates vessel wall elasticity with hemodynamics, enabling the calculation of cardiac output (CO) under physiological conditions [21]. This model has been validated in studies calculating CO and cerebral blood flow (CBF) in older adults [22,25], but a similar model has never been applied specifically to POTS before. Our approach thus demonstrates innovation through its comprehensive integration of blood volume, vascular stiffness, and autonomic feedback mechanisms. Additionally, advanced neuroimaging methods, like functional magnetic resonance imaging, are being employed to explore brain activity and ANS function in POTS patients, providing new means for investigating POTS mechanisms [26].

This research is intended to explore and analyze the contributions of hypovolemia, vascular dysfunction, and ANS dysregulation to the pathogenesis of POTS using a novel hemodynamic model. By doing so, we seek to provide new theoretical insights to support the diagnosis and treatment of this condition.

The abbreviations used in this paper are listed in Table 1.

**Table 1:** List of abbreviations used in this paper.

Abbreviation	Full Name
ANS	Autonomic nervous system
BP	Blood pressure
CBF	Cerebral blood flow
CO	Cardiac output
DBP	Diastolic blood pressure
HR	Heart rate
POTS	Postural Orthostatic Tachycardia Syndrome
RHR	Resting heart rate
SBP	Systolic blood pressure
SEDI	Strain energy density increment
SNS	Sympathetic nervous system

## 2 Methods and clinical data

### 2.1 A novel fluid-structure interaction model of blood circulation flow

**Fig 1:**
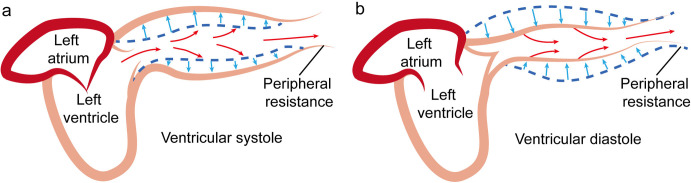
Schematic diagram of aortic wall elasticity. Notes: a) Ventricular systole, b) Ventricular diastole.

The fluid-structure interaction model for blood circulation, put forward by Cheng et al., offers a fresh perspective beyond the Windkessel model [21]. This model emphasizes the initial kinetic energy created by the heart’s pumping motion and supposes that the potential energy (i.e., strain energy) inside the arteries plays a dominant role in blood flow. During ventricular systole, approximately 2/3 of the blood ejected from the left ventricle is stored temporarily in the aorta due to peripheral resistance [27]. In the phase of cardiac diastole, the ejection of blood comes to a halt. At this time, the strain energy accumulated in the elastic arterial vessels drives the blood to keep flowing. Fig 1 schematically demonstrates this principle: during systole (Fig 1a), the aorta expands and stores energy as blood is ejected; during diastole (Fig 1b), the aortic wall recoils, propelling blood forward even in the absence of cardiac pumping. This highlights the role of aortic elasticity in continuous blood circulation.

In applying this model to our study, we characterize the strain energy density increment (SEDI) using measurable physiological variables and reasonable assumptions. Prior research indicates that within the physiological range, the mechanical behavior of the aorta can be approximated as linear elasticity. The ascending aorta was considered as a thin-walled cylinder [28,29]. According to the circumferential stress-strain relationship, the SEDI of the elastic blood vessels during diastole and systole can be approximated as:


$$\Delta u=\frac{\lambda_{z}^{2}\varphi^{2}{\left(\alpha_{s}p_{s}-\alpha_{d}p_{d}\right)}^{2}}{8E}$$ (1)

Δ*u* refers to the SEDI of the aorta. The axial pre-stretch ratio *λ*_*z*_ is defined as the quotient of the *in situ* length *l* to the *in vitro* length *l*_*0*_. *φ* serves as the constraint coefficient of the aorta. *α*_*d*_ and *α*_*s*_ are the coefficients, with *α*_*d*_* *= *D*_*d*_/*h*_*d*_ and *α*_*s*_* *= *D*_*s*_/*h*_*s*_, *E* represents the circumferential elasticity modulus of the aortic vessel. *D*_*d*_*,*.*D*_*s*_, *h*_*d*_ and *h*_*s*_ respectively stand for the systolic and diastolic inner diameters and wall thicknesses of the aorta. The symbols *p*_*d*_ and *p*_*s*_ signify diastolic and systolic BP.

Turbulence in large arteries (e.g., the aortic root) is expected only when CO is extremely high, such as during strenuous exercise [30,31]. In the absence of evidence for sustained turbulence in human arteries at rest [32], arterial blood flow is assumed to be laminar under normal conditions [33,34]. In the present study, aortic blood flow is treated as laminar.

Case of systolic and diastolic blood kinetic energy increments:


$$\Delta K=\frac{\varphi \gamma \left(\alpha_{s}p_{s}-\alpha_{d}p_{d}\right)V^{2}}{E}$$ (2)

where *φ* stands for the constraint coefficient of the aorta, *γ* represents the weight per unit volume. *V* is the flow distance per unit weight of blood per pulse across the arterial cross-section, which is defined as the characteristic velocity.

The dissipated energy increment of fluid flow, indicated by Δ*w*_*τ*_, representing the increment of work performed by blood because of wall shear stress, is estimated in the following way:


$$\Delta w_{\tau }=-\lambda_{z}\varphi \gamma \lambda \left(D_{s}-D_{d}\right)K^{2}V^{2}$$ (3)

in this context, *λ* serves as the friction loss factor of the elastic vessel. It depends on flow conditions, blood lipid levels, and fluid viscosity. For laminar flow, *λ* is computed as *λ = *64*/Re*, with *Re* being the Reynolds dimensionless number (*Re* = *VD/μ*), μ denotes blood viscosity, and *K* is a coefficient. According to the Hamilton principle of fluid-structure coupling, the functional (*χ*) of the constructed aortic vessel per pulse unit length can be represented as follows.


$$\chi =∭\frac{\lambda_{z}^{2}\varphi \gamma \left(\alpha_{s}p_{s}-\alpha_{d}p_{d}\right)V^{2}}{E}dV-∭\frac{\varphi^{2}{\left(\alpha_{s}p_{s}-\alpha_{d}p_{d}\right)}^{2}}{8E}dV-\iint \lambda_{z}\left(D_{s}-D_{d}\right)\varphi \gamma \lambda K^{2}V^{2}dS$$ (4)

The *V* can be acquired via the variational operation of *D*_*d*_ and *D*_*s*_.


$$V=\lambda_{z}\sqrt{\frac{\varphi h}{D\gamma (1+\beta )}\left(\alpha_{s}p_{s}-\alpha_{d}p_{d}\right)}$$ (5)

Where


$$\beta =\frac{4E\lambda K^{2}(h_{d}-h_{s})}{D\left(p_{s}-p_{d}\right)\varphi }$$ (6)

*β* is designated as the coupled energy dissipation coefficient. This coefficient is impacted by vessel geometry, pulse pressure, lipid levels, vessel stiffness, and fluid viscosity. Consequently, the average blood flow *Q* per cardiac cycle through the cross-section can be expressed as:


$$Q=Q_{0}+\Delta Q=\lambda_{z}\left(1+\varphi \frac{Dp}{Eh}\right)A_{f}\sqrt{\frac{\varphi h}{D\gamma (1+\beta )}\left(\alpha_{s}p_{s}-\alpha_{d}p_{d}\right)}$$ (7)

*A*_*f*_ represents the initial cross-sectional area of blood flow in the aorta. The average flow rate per minute through the arterial cross-section *Q̅* is calculated as follows:


$$\overset{―}{Q}=n\lambda_{z}\left(1+\varphi \frac{Dp}{Eh}\right)A_{f}\sqrt{\frac{\varphi h}{D\gamma (1+\beta )}\left(\alpha_{s}p_{s}-\alpha_{d}p_{d}\right)}$$ (8)

In this equation, *n* refers to the pulse frequency. The model grasps the coupled influences of various physiological variables on blood supply. It presents an approximate analytical solution for the mean flow rather than a numerical one.

### 2.2 Existing clinical records

In order to thoroughly investigate the pathophysiological mechanisms of POTS, the current study synthesized and analyzed clinical data from a range of relevant literature. These data comprehensively encompassed several crucial parameters of the cardiovascular system, such as HR, BP, blood volume, ANS function, vascular function and CBF. These clinical records serve as the foundation for a profound comprehension of the pathomechanisms of POTS. Moreover, they offer essential support for subsequent hemodynamic calculations and analyzes.

#### 2.2.1 HR and BP.

For normal adult, the systolic blood pressure (SBP) is less than 120 mmHg and diastolic blood pressure (DBP) is less than 80 mmHg [35]. The resting heart rate (RHR) typically falls within the range of 60–100 bpm [36].

**Fig 2:**
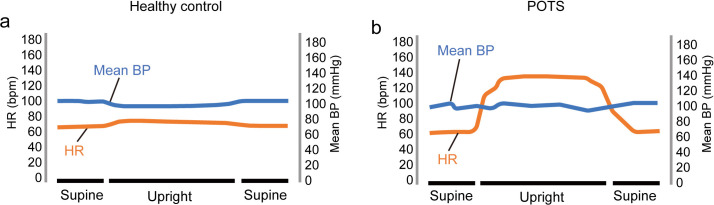
Supine and upright HR and BP schematic diagram. Notes: Supine and upright HR (orange) and BP (blue) profiles of healthy controls (left panel) and POTS patients (right panel) demonstrating an exaggerated HR increase (>30 bpm) and relatively stable BP.

The mean BP and HR changes in healthy controls and POTS patients during changes in body position are shown in Fig 2 [1,3,37–40]. Among healthy individuals, upon changing from the supine to the upright posture, neither the mean BP nor HR experiences a notable change. In contrast, in POTS patients, there is a significant change in HR due to their own feedback regulation, especially an increase in SNS activity, while there is no significant change in the mean BP during changes in body position [1]. Based on the primary diagnostic criteria for POTS, when patients stand up, their HR increases by at least 30 bpm, along with symptoms like fatigue, palpitations, and dizziness [2,4,39].

#### 2.2.2 Blood volume.

Patients with POTS commonly exhibit a blood volume reduction ranging from 10% to 30% [41]. Additionally, compared to subjects with normal blood volume, POTS patients exhibit relatively lower plasma renin activity and aldosterone levels upon standing [41].

A decrease in blood volume usually brings about a decline in BP. As blood volume diminishes, the quantity of blood returning to the heart (venous return) lessens, leading to a decrease in CO. This reduction in CO gives rise to a fall in arterial BP, especially a substantial drop in systolic BP. To compensate for this, the body increases peripheral vascular resistance through vasoconstriction in an effort to maintain BP. Consequently, patients with low blood volume often exhibit lower BP than normal individuals [42].

#### 2.2.3 Vascular dysfunction.

Vascular function alterations, lead to hemodynamic abnormalities that are pivotal in the pathogenesis of POTS [43–45]. Increased plasma norepinephrine levels are associated with abnormal vascular function, which may contribute to POTS [46]. Vascular dysfunction can interfere with normal vascular contraction, thereby preventing adequate blood return to the heart.

#### 2.2.4 Abnormal ANS function.

During autonomic function tests, POTS patients generally have normal autonomic reflexes. For example, the sinus arrhythmia ratios in response to deep breathing remain intact, and they show a strong stress response during the Valsalva Maneuver [16]. Usually, POTS patients experience a substantial rise in HR during the upright tilt test. This is a physiological reaction aimed at maintaining arterial pressure when venous blood pools [2]. Both hypovolemia and vascular dysfunction causing POTS involve abnormal regulation of ANS.

#### 2.2.5 CBF.

CBF varies significantly among different populations, influenced by factors such as race, age, and gender. Under typical circumstances, the CBF necessary to sustain normal neurological function is estimated to be approximately 50–55 mL/100 g of brain tissue per minute [47]. According to the findings of P. Scheel et al. [48], the reference values of CBF in healthy adults aged 20–39 years are 727 ± 102 mL/min, and CBF in this range ensures that the brain receives sufficient oxygen and nutrients to support its normal physiological activities.

### 2.3 Model input parameters

To investigate the pathological mechanisms of POTS, a blood circulation fluid-solid coupling model was used to analyze both healthy controls and pathological conditions, including hypovolemia and vascular dysfunction, based on Eq 8.

For the healthy control group, SBP/DBP was set to 120/80 mmHg [35], with a HR of 60 bpm [36]. The circumferential elastic modulus of the ascending aorta was set to 0.4 MPa based on existing literature [49].

Clinical data indicate that hypovolemia-induced POTS is characterized by a modest reduction in arterial pressure at rest and a pronounced tachycardic response to standing [42,50]. Accordingly, we adopted supine values of SBP/DBP = 110/75 mmHg with HR = 60 bpm, and orthostatic values of SBP/DBP = 100/75 mmHg with HR = 100 bpm. Clinical data show that POTS patients with endothelial or arterial dysfunction maintain near-normal blood pressure but develop marked tachycardia on standing [10]. Therefore, we assigned supine values of SBP/DBP = 120/80 mmHg and HR = 60 bpm, and orthostatic values of SBP/DBP = 110/70 mmHg and HR = 100 bpm.

To reproduce the loss of arterial compliance reported in these cohorts, the circumferential elastic modulus (*E*) of the ascending aorta was increased from 0.4 MPa (healthy control) to 0.6 MPa in the supine state and to 0.8 MPa during orthostasis. This rise lies within the stiffness elevations measured by ultrasound and magnetic resonance imaging in POTS patients who exhibit endothelial dysfunction and increased pulse-wave velocity [10,43,44].

The dimensions of the ascending aorta are influenced by factors such as age, sex, and body size [51–53]. Current research has determined the average internal diameter of the ascending aorta to be 29.4 ± 3.7 mm [54], and the median wall thickness in women to be 1.46 mm [29]. Consequently, the internal diameter and wall thickness of the ascending aorta are set at a pressure of 80 mmHg. Given the assumption that the arterial wall volume remains constant within the physiological deformation range, it can be considered an incompressible material [55,56], thus allowing for the calculation of wall thickness under other pressure conditions.

By substituting the corresponding BP, HR, and other parameters into Eq 8, the analytical solution for reduced CO can be derived, rather than a numerical approximation. In healthy individuals, approximately 14% of the CO is directed to the brain via the bilateral internal carotid arteries and vertebral arteries [57].

## 3 Results

Employing Eq 8, we quantitatively assessed hemodynamic parameters in the healthy control cohort and patients with two distinct subtypes of POTS, with all results systematically tabulated in Table 2.

**Table 2:** Estimation of the CO and CBF.

	Healthy control		Hypovolemia-induced POTS		Vascular dysfunction-induced POTS	
	Supine position	Orthostatic position	Supine position	Orthostatic position	Supine position	Orthostatic position
*P*_*s*_ (mmHg)	120	120	110	100	120	110
*P*_*d*_ (mmHg)	80	80	70	70	80	80
*P* (mmHg)	93.33	93.33	83.33	80.00	93.33	90.00
*λ*_*z*_	1.20	1.20	1.20	1.20	1.20	1.20
*ε*_*Ap*_	0.06	0.06	0.04	0.02	0.04	0.03
*D*_*so*_ (mm)	33.15	33.15	32.69	32.41	32.88	32.72
*h*_*s*_ (mm)	1.42	1.42	1.44	1.46	1.43	1.44
*D*_*s*_ (mm)	30.31	30.31	29.80	29.50	30.01	29.84
*α*_*s*_	21.35	21.35	20.65	20.26	20.94	20.72
*D*_*do*_ (mm)	32.32	32.32	32.14	32.14	32.32	32.32
*h*_*d*_ (mm)	1.46	1.46	1.47	1.47	1.46	1.46
*D*_*d*_ (mm)	29.40	29.40	29.20	29.20	29.40	29.40
*α*_*d*_	20.14	20.14	19.86	19.86	20.14	20.14
*E* (MPa)	0.4	0.4	0.4	0.4	0.6	0.8
*Re*	1000	1000	1000	1000	1000	1000
*β*	0.60	0.60	0.48	0.70	0.65	1.24
*V* (m)	0.13	0.13	0.12	0.06	0.12	0.06
*Q* (L/bpm)	0.08	0.08	0.07	0.04	0.07	0.04
*n* (bpm)	65	65	65	105	65	105
CO (L/min)	5.12	5.12	4.63	3.94	4.65	4.01
CBF (mL/min)	717	717	649	551	651	562

Notes: *P*_s_ and *P*_d_ represent the systolic and diastolic BP, respectively; *P* denotes the mean BP; λ_z_ is the axial pre-stretch ratio; ε_Ap_ represents the strain of the cross-sectional area; *D*_so_ is the outer diameter of the artery during systole; *h*_s_ is the arterial wall thickness during systole; *D*_s_ is the inner diameter of the artery during systole; α_s_ is the ratio of *D*_s_ to *h*_s_ (α_s_ = *D*_s_/*h*_s_); *D*_do_ is the outer diameter of the artery during diastole; *h*_d_ is the arterial wall thickness during diastole; *D*_d_ is the inner diameter of the artery during diastole; α_d_ is the ratio of *D*_d_ to *h*_d_ (α_d_ = *D*_d_/*h*_d_); *E* is the circumferential elastic modulus of the artery; *Re* is the Reynolds number; β is the coupled energy dissipation coefficient; *V* is the characteristic velocity of blood flow; *Q* is the blood volume passing through the cross-section per stroke; *n* is the HR; CO is the cardiac output; CBF is the cerebral blood flow, calculated as CBF = CO × 14%.

During the process of postural changes, the physiological parameters of the healthy control group demonstrate stability. Specifically, the BP, HR, and CBF of the healthy control group remain stable before and after transitioning from a supine to an upright position (see calculations for the healthy control group in Table 2). This finding suggests that the cardiovascular system of healthy individuals possesses the capacity to effectively adapt to changes in posture, thereby maintaining the balance of physiological parameters. In contrast, POTS patients exhibit minimal changes in BP but a significant increase in HR (an increase of 35 bpm) during the transition from supine position to orthostatic position. Conversely, POTS patients demonstrate a decline in CBF of approximately 100 mL/min in the upright position (see calculations for POTS patients in Table 2).

**Fig 3:**
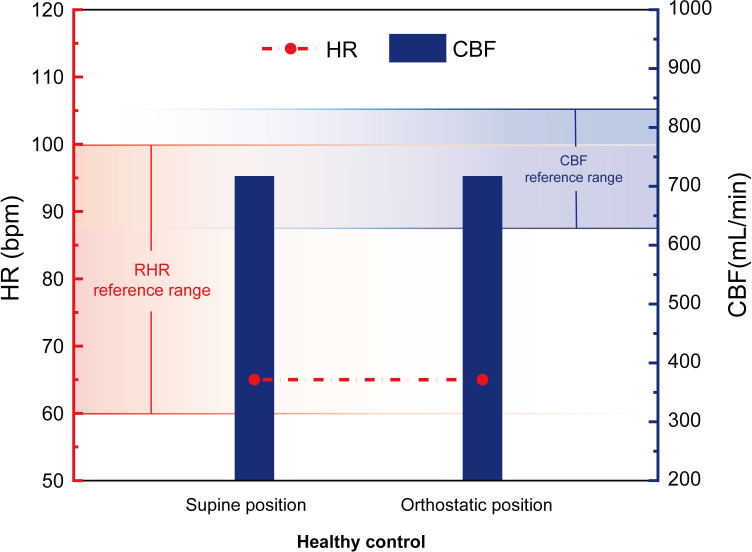
Healthy control group HR and CBF changes.

The HR and CBF during the two physiological phases before and after the postural change are shown in Fig 3.

Fig 3 indicates the reference range for RHR in normal adults, which is 60–100 bpm [36], and the reference range for CBF in healthy adults aged 20–39, as reported by Scheel et al., which is 727 ± 102 mL/min [48]. The HR and CBF values of the healthy control group fall within these respective reference ranges.

**Fig 4:**
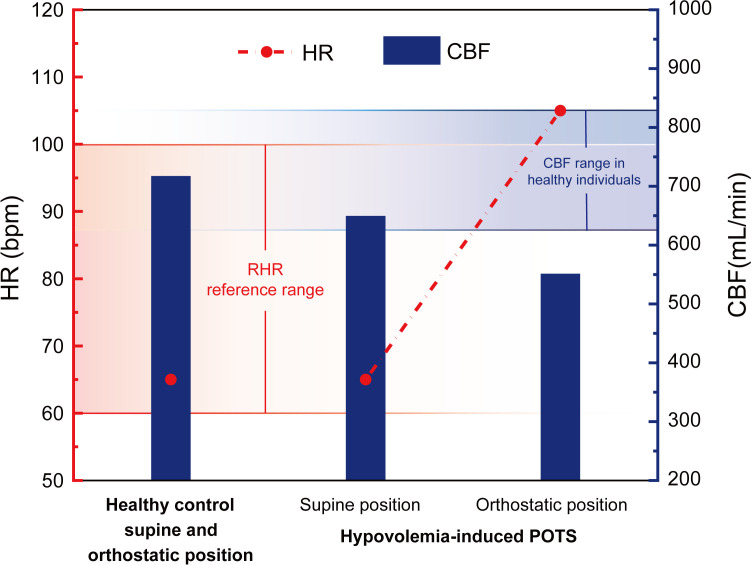
Healthy control group HR and CBF, hypovolemia-induced POTS HR and CBF changes.

Based on the calculated data for hypovolemia-associated POTS in Table 2, the changes in HR and CBF associated with hypovolemia-induced POTS are illustrated in Fig 4.

As demonstrated in Fig 4, the reference range for the RHR in healthy adults is 60–100 bpm [36], and the CBF reference range for individuals aged 20–39, as reported by Scheel et al., is 727 ± 102 mL/min [48]. In patients diagnosed with POTS, the CBF while in a supine position is 682 mL/min, which falls within the reference range but is lower than that observed in the healthy control group (as shown in Fig 4). During orthostatic conditions, the ANS increases the HR by 40 bpm, while SBP and DBP remain relatively stable, with a reduction in pulse pressure by 10 mmHg. Consequently, CBF decreases to 551 mL/min. The elevated HR exceeds the reference range.

**Fig 5:**
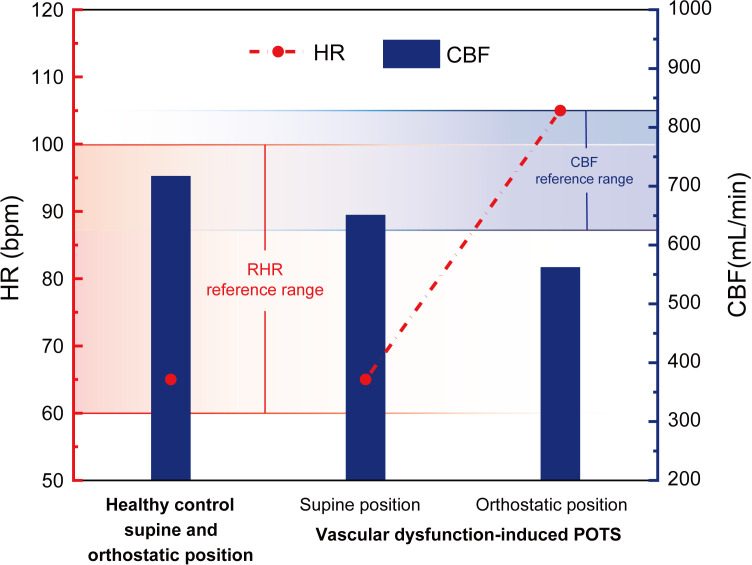
Healthy control group HR and CBF, vascular dysfunction-induced POTS HR and CBF changes.

Based on the calculated data for vascular dysfunction-induced POTS in Table 2, the changes in HR and CBF due to vascular dysfunction-induced POTS are illustrated in Fig 5.

As demonstrated in Fig 5, the reference range for the RHR in healthy adults is 60–100 bpm [36], and the CBF reference range for individuals aged 20–39, as reported by Scheel et al., is 727 ± 102 mL/min [48]. In POTS patients, the CBF in the supine position is 686 mL/min, which falls within the reference range but is lower than that of the healthy control group (as shown in Fig 5). During orthostatic conditions, the ANS increases the HR by 40 bpm, while SBP and DBP remain relatively stable, exhibiting a decline in pulse pressure by 10 mmHg. Consequently, CBF decreased to 562 mL/min. The elevated HR exceeds the reference range.

## 4 Discussion

The findings of this study align with existing clinical research: the association between hypovolemia, cerebral hypoperfusion, and increased heart rate has been widely recognized [6,58]. Furthermore, the significance of vascular dysfunction in POTS has been verified by a variety of studies [43–45], alongside ANS dysfunction [7], all of which are critical pathological factors in POTS. In contradistinction to conventional medical research methodologies, such as Mendelian randomization [59] and meta-analysis [60], this study employs a computational model to quantitatively analyze hemodynamic parameters, thereby enabling a precise evaluation of the roles of blood volume, vascular function, and the ANS in POTS.

### 4.1 Analysis for healthy control group

For the healthy control group transitioning from supine position to orthostatic position, the body employs neural reflexes and cardiovascular regulatory mechanisms to induce vasoconstriction while gradually enhancing myocardial contractility, thereby maintaining BP and relatively stable CO [61]. This rapid compensatory mechanism ensures that BP and HR values in the healthy control group typically exhibit minimal changes.

### 4.2 Hypovolemia-induced POTS

Hypovolemia is a principal pathophysiological cause in POTS patients. When peripheral resistance remains constant, a decline in blood volume is shown as a drop in BP. Changing from the supine to the upright posture can cause a shortage of CO, thus lowering CBF [2,8]. Under these conditions, the ANS compensates for the inadequate cerebral blood supply by increasing HR, resulting in abnormally elevated HR.

The elevated HR exceeds the reference range, leading to symptoms such as palpitations. Compared to the CBF reference range, POTS patients exhibit insufficient myocardial blood supply and reduced CBF as shown in Fig 4), resulting in symptoms such as dizziness. These findings are consistent with existing clinical studies [42,50].

As demonstrated in Section 2.2.2, clinical records indicate that patients with low blood volume frequently exhibit lower BP and a diminished pulse pressure. SEDI is directly associated with pulse pressure (see Eq 1); therefore, a smaller pulse pressure leads to a lower SEDI. Further, based on Eq 8, a decrease in SEDI along with a decrease in BP leads to a reduction in the flow through the arterial cross-section (*Q̅*). This directly diminishes the blood flow to the myocardium and CBF.

In cases of hypovolemia, transitioning from a supine to an upright position leads to insufficient blood supply, reducing the blood flow to the brain. To compensate for this inadequate cerebral blood supply, the body increases HR through the feedback regulation mechanism of the ANS, in an attempt to maintain sufficient cerebral blood flow. However, even with an increased HR, the CBF remains insufficient to meet the brain’s demand for oxygen and nutrients. This is the pathological mechanism by which hypovolemia triggers POTS.

### 4.3 Vascular dysfunction-induced POTS

Patients with POTS may exhibit a decrease in vascular compliance [10]. Although both venous and arterial compliance can be decreased in POTS, this study’s calculations involve only changes in aortic compliance. In the aorta, this manifests as an increase in the circumferential elastic modulus (*E*), which reduces the strain energy stored during cardiac systole and thereby diminishes the aorta’s ability to propel blood flow. During orthostatic alterations, this leads to blood accumulating in the lower extremities and a decline in the amount of venous blood returning to the heart. These physiological changes precipitate a compensatory increase in HR [62].This compensatory tachycardia often causes palpitations, and the resulting cerebral hypoperfusion leads to dizziness – consistent with clinical observations [43–45].

Normally, the body maintains adequate CO and BP by regulating vascular tone and causing vasoconstriction in the lower extremities to return blood to the heart. As demonstrated in Section 2.2.3, alterations in vascular function, such as increased vascular stiffness, have the capacity to interfere with this process. Eq 1 demonstrates that an increase in the circumferential elastic modulus *E* will inevitably result in a decrease in SEDI. Eq 8 indicates that an increase in the circumferential elastic modulus *E* of the vessel will inevitably lead to a decrease in CO, which in turn results in a CBF deficit.

Upon standing, a patient with vascular dysfunction experiences excessive blood pooling in the lower limbs due to gravity. Because stiff vessels cannot effectively return blood to the heart, the autonomic reflex raises HR – yet cerebral blood flow remains insufficient. This sequence underlies POTS in the setting of vascular dysfunction.

### 4.4 Role of autonomous feedback regulation

Abnormal ANS function is essential in the pathogenesis of POTS. The carotid sinus and aortic arch perceive changes in BP and convey signals to the central nervous system. The CNS causes increased heart rate by regulating sympathetic and parasympathetic activity, resulting in sympathetic excitation and parasympathetic inhibition.

As demonstrated in Table 2, the calculations reveal that an imbalance in autonomic feedback regulation can lead to a pathological elevation of HR. This finding aligns with the clinical observation that POTS patients typically manifest a pronounced HR increase during postural changes [2,40,63]. This result emphasizes the importance of factoring in the regulatory function of ANS during POTS treatment. Here, the balance between SNS and parasympathetic nervous system can be regulated by means of medications or other interventions. Without the increase in HR due to autonomous feedback regulation, patients may present with orthostatic hypotension. These cases indicate that ANS feedback regulation confirm the pivotal role in the pathophysiology of POTS.

In addition, when the body is in a state of hypovolemia, the adrenergic system becomes activated, triggering the release of catecholamines including epinephrine and norepinephrine. These hormones stimulate *β*-adrenergic receptors in the heart, thereby accelerating HR [62].

The brain accounts for 20% of the body’s total metabolic energy expenditure, and maintaining CBF is crucial for preserving brain function [46]. During the pathology of POTS, the body attempts to maintain CBF through its own feedback regulatory mechanisms, including the ANS. Particularly, as a person moves from a supine to an upright stance, blood has a tendency to gather in the lower limbs because of gravity, which brings about a decline in CBF. To compensate for this decline, the ANS increases HR by enhancing sympathetic nerve activity and adrenaline levels, as shown in Eq 8. The increase in HR reflects the activation of the ANS and elevated adrenaline levels, which is a physiological compensatory response designed to elevate the CBF supply by increasing the frequency of heart contractions.

Although a higher heart rate increases arterial flow *Q̅*, in POTS this compensation remains inadequate for cerebral needs. The result is a mismatch: an excessive HR rise with still insufficient cerebral perfusion. This imbalance explains common POTS symptoms (dizziness, weakness, palpitations) and is a key feature of POTS pathophysiology.

### 4.5 Rationality of current treatment approaches

Combined with the above hemodynamic analysis, the rationality of clinical treatment strategies for POTS is discussed as follows:

**Blood volume management**: The findings emphasize the necessity of evaluating and managing patients’ blood volume. Clinicians are advised to consider increasing salt intake or using water retention agents as a treatment strategy to improve hypovolemia and thereby alleviate POTS symptoms [12].
**Physical therapy and vascular elasticity improvement**: In addition to using compression stockings to reduce venous pooling, patients can improve arterial elasticity through exercise, diet, or medications [64,65]. Such measures enhance blood distribution during standing and lessen the reflex tachycardia.
**ANS modulation therapy**: Given the role of abnormal ANS function in POTS, medications such as *β*-blockers (e.g., metoprolol) may be considered to control excessive heart rate increases [20,66,67]. Additionally, progressive postural training has been shown to be an effective non-pharmacological approach that enhances patients’ adaptation and tolerance to postural changes.
**Comprehensive treatment strategy**: Given the complexity of POTS, a multimodal treatment approach is necessary, including medication, physical therapy, psychological support, and lifestyle adjustments, to achieve the best therapeutic outcomes.

## 5 Limitations

This study is based on a theoretical computational model using parameters derived from existing literature and assumes normal blood viscosity and hematocrit, thus limiting its individual applicability. Additionally, the estimation that cerebral blood flow constitutes approximately 14% of CO was primarily derived from data on fit men, which may differ in predominantly female POTS populations. However, as our analysis mainly focused on relative changes, we believe the conclusions regarding cerebral blood flow trends remain valid.

## 6 Conclusion

This study elucidated the pathomechanisms of POTS using a coupled hemodynamic model. The computations showed that insufficient blood volume and abnormal vascular function are the contributors to heart rate abnormalities in POTS patients. These factors result in reduced cerebral blood supply, thereby triggering a compensatory tachycardia via autofeedback regulation. The findings of this study offer novel theoretical insights into the diagnosis and management of POTS, underscoring the significance of a holistic treatment approach. This approach encompasses blood volume management, physical therapy, ANS modulation therapy, and lifestyle adjustments, all aimed at optimizing treatment efficacy.
